# Development and validation of a deep learning-based model to distinguish acetabular fractures on pelvic anteroposterior radiographs

**DOI:** 10.3389/fphys.2023.1146910

**Published:** 2023-04-28

**Authors:** Pengyu Ye, Sihe Li, Zhongzheng Wang, Siyu Tian, Yi Luo, Zhanyong Wu, Yan Zhuang, Yingze Zhang, Marcin Grzegorzek, Zhiyong Hou

**Affiliations:** ^1^ Third Hospital of Hebei Medical University, Shijiazhuang, Hebei, China; ^2^ University of Lübeck, Lübeck, Schleswig-Holstein, Germany; ^3^ Heidelberg University, Heidelberg, Baden-Württemberg, Germany; ^4^ Orthopedic Hospital of Xingtai, Xingtai, China; ^5^ Xi’an Honghui Hospital, Xi’an, Shaanxi, China

**Keywords:** deep learning, acetabular fracture, pelvic anteroposterior radiograph, DenseNet, diagnosis

## Abstract

**Objective:** To develop and test a deep learning (DL) model to distinguish acetabular fractures (AFs) on pelvic anteroposterior radiographs (PARs) and compare its performance to that of clinicians.

**Materials and methods:** A total of 1,120 patients from a big level-I trauma center were enrolled and allocated at a 3:1 ratio for the DL model’s development and internal test. Another 86 patients from two independent hospitals were collected for external validation. A DL model for identifying AFs was constructed based on DenseNet. AFs were classified into types A, B, and C according to the three-column classification theory. Ten clinicians were recruited for AF detection. A potential misdiagnosed case (PMC) was defined based on clinicians’ detection results. The detection performance of the clinicians and DL model were evaluated and compared. The detection performance of different subtypes using DL was assessed using the area under the receiver operating characteristic curve (AUC).

**Results:** The means of 10 clinicians’ sensitivity, specificity, and accuracy to identify AFs were 0.750/0.735, 0.909/0.909, and 0.829/0.822, in the internal test/external validation set, respectively. The sensitivity, specificity, and accuracy of the DL detection model were 0.926/0.872, 0.978/0.988, and 0.952/0.930, respectively. The DL model identified type A fractures with an AUC of 0.963 [95% confidence interval (CI): 0.927–0.985]/0.950 (95% CI: 0.867–0.989); type B fractures with an AUC of 0.991 (95% CI: 0.967–0.999)/0.989 (95% CI: 0.930–1.000); and type C fractures with an AUC of 1.000 (95% CI: 0.975–1.000)/1.000 (95% CI: 0.897–1.000) in the test/validation set. The DL model correctly recognized 56.5% (26/46) of PMCs.

**Conclusion:** A DL model for distinguishing AFs on PARs is feasible. In this study, the DL model achieved a diagnostic performance comparable to or even superior to that of clinicians.

## Introduction

Acetabular fractures (AFs) are one of the most complex traumas treated by orthopedic surgeons, which are mostly caused by high-energy trauma and have complicated and variable imaging features (Ziran et al., 2019). They are regularly accompanied by other concomitant injuries and occur in approximately 3–8 cases per 100,000 people (Laird and Keating, 2005; Melhem et al., 2020; Lundin et al., 2021). In addition, the hip joint is an essential weight-bearing joint with a wide range of motion, and its injury has a significant detrimental effect on an individual’s physical function, ability to work, and participation in social activities (FFA et al., 2021). Hence, AFs must be accurately and promptly detected, since diagnostic failure can lead to hip instability, post-traumatic osteoarthritis, and other poor outcomes (Scheinfeld et al., 2015).

A pelvic anteroposterior radiograph (PAR) provides visualization of the continuity and integrity of the bones in the pelvic and upper femur regions. It is the first choice for screening AFs (Trauma, 2018; Kitamura, 2020). Generally, traumas such as AFs are initially diagnosed and tended to by young trauma orthopedic surgeons because of their emergency nature (Oka et al., 2021). However, the diagnosis of AFs on X-ray images is difficult because AFs are uncommon, the femoral head and acetabulum overlap, and the potential existence of a single smaller fragment (Scheinfeld et al., 2015). Furthermore, radiologists are not always readily available, particularly in local hospitals or rural areas, which increases the uncertainty of diagnosis (Cheng et al., 2021; Zech et al., 2022). Thus, a new solution for the accurate and rapid detection of AFs is urgently required.

Deep learning (DL) is a group of artificial intelligence techniques that enable algorithms to learn from input data, identify features, and classify data after multiple iterations (Kuo et al., 2022; Zech et al., 2022). DL has evolved in leaps and bounds in the last few years and has been increasingly applied in radiology, orthopedics, and traumatology (Jones et al., 2020; Kuo et al., 2022). Previous studies have shown that fracture detection using DL can be on par with or exceed the diagnostic performance of physicians (Yamada et al., 2020; Cheng et al., 2021; Kuo et al., 2022). Although the detection of hip and pelvic ring fractures using DL on PAR has been reported (Kitamura, 2020; Yamada et al., 2020; Cheng et al., 2021; Liu et al., 2022), to our knowledge, studies focusing on identifying AFs are lacking. This study aimed to develop a DL model for detecting AFs on PAR and validate the strength of the model by comparing its diagnostic performance with that of orthopedic clinicians.

## Materials and methods

This study was approved by the institutional review board. The requirement for informed consent was waived owing to the retrospective nature of the study.

### Data selection

Consecutive patients with AFs who underwent PAR from January 2013 to October 2021 at a big level I trauma center were retrospectively reviewed. Moreover, AF patients from two external hospitals from January 2020 to December 2020 were selected as validation data. Images were collected from a picture archiving and communication system (PACS). The inclusion criteria were as follows: 1) AFs with a clear history of trauma, 2) adults (>18 years old), and 3) the first PAR was taken after the patient arrived at our hospital. The exclusion criteria were as follows: 1) pathological fractures; 2) congenital hip malformations; 3) poor image quality or foreign bodies occluding the acetabulum. More advanced imaging examination evidence from PACS, such as CT, MRI (if any), and clinical information from patient files provide sufficient support for the diagnosis and classification of AFs.

Demographic characteristics, including age, sex, injured side, and mechanism of injury, were recorded from patient files. Two traumatic orthopedic surgeons evaluated the fracture type (one with 6 years of experience and the other with 10). When an inconsistency occurred, they discussed this and made a final decision with another orthopedic specialist with more than 25 years of experience. All AFs were classified into three main types: type A, type B, and type C, based on the “Three-column classification of AFs” (Zhang et al., 2019). According to the above classification, types A, B, and C represent fractures involving one, two, and three columns of the acetabulum, respectively, and indicate more severe and complicated fractures.

### Data preprocessing

**FIGURE 1:**
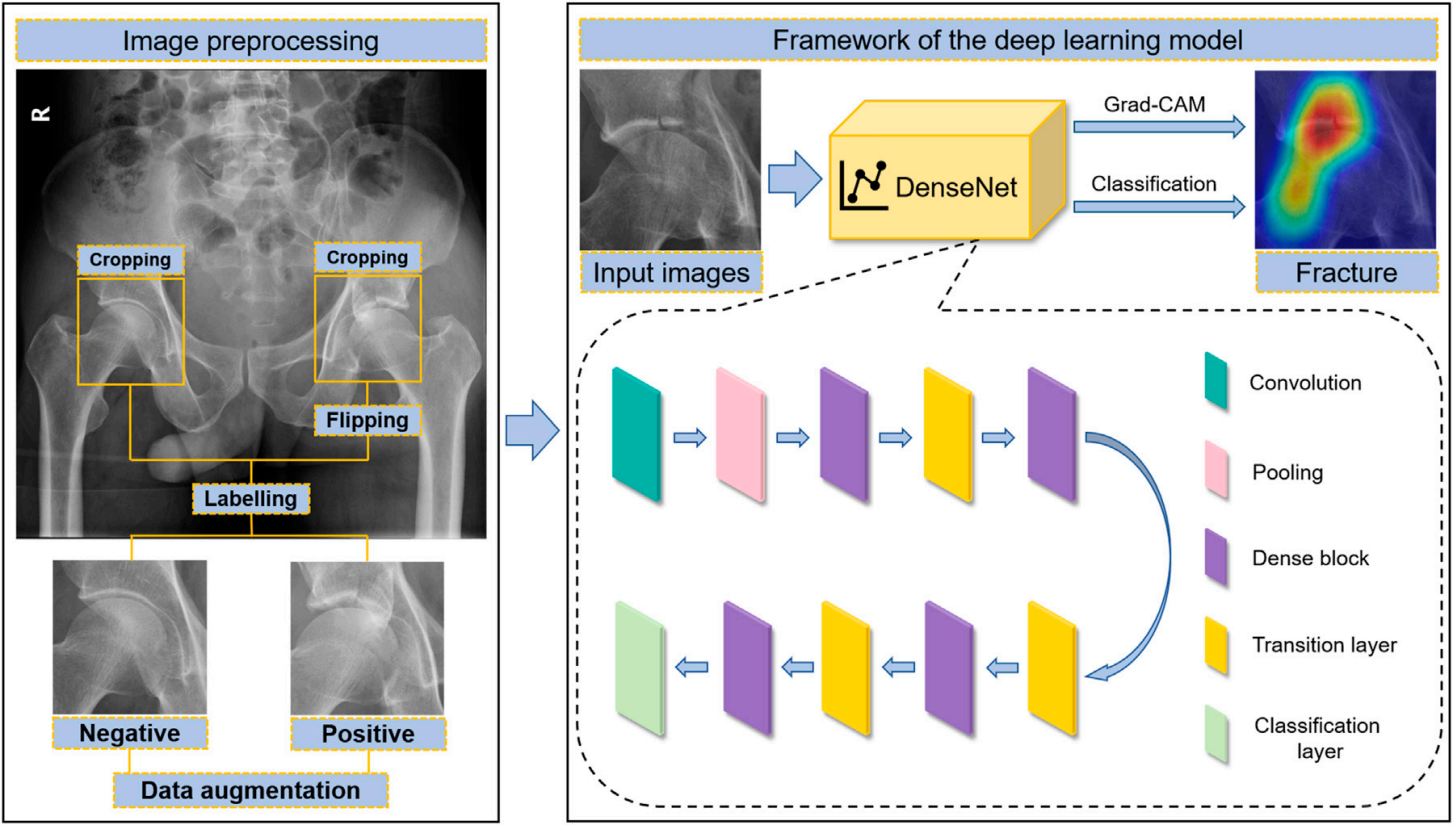
The workflow of acetabular fracture detection by artificial intelligence, including image preprocessing and the framework of the DL model.

The original panoramic images of pelvic radiographs were obtained from the PACS with sizes of 1,024 × 1,024 pixels and 32-bit depth. The regions of interest (ROIs) containing the acetabulum were manually cropped using a 256 × 256 square bounding box with Snipaste software (v2.4.0-Beta) and converted to 8-bit depth grayscale images to match the predefined input image of the DL model. Both sides of the acetabulum were extracted individually and labelled as normal (negative) or not (positive). Moreover, because the left and right acetabula are symmetrical, the cropped left acetabular images were flipped horizontally and used as right acetabular developing images (Figure 1). DL often requires a lot of data to achieve satisfactory results. Data augmentation is a commonly used data preprocessing technique in DL that can increase the quantity of data for training. To improve the robustness of the model, the development dataset was augmented 15 times by brightness control, rotation, blurring, and noise addition. Details of the data augmentation are provided in the Supplementary Material.

### Model architecture and developing

Our DL network model was built based on DenseNet-169 architecture (Huang et al., 2017). DenseNet introduced dense connections to extract features from images. Compared with other convolutional networks, it can alleviate the vanishing-gradient problem, reduce the complexity of the model, and implement feature reuse (Huang et al., 2017). Our model was mainly composed of a 7 × 7 convolutional layer, followed by a max pooling layer, four dense blocks, three transition layers between dense blocks, and a classification layer at the end. Inside each dense block, 1 × 1 convolutional bottleneck layers and 3 × 3 convolutional layers were connected to each other in series. Pre-activation was introduced to each convolutional layer. The final classification layer was constructed using a fully connected layer, followed by sigmoid calculation. The output of the complete model was described as the probability of indicating the result of the prediction (Supplementary Table S1). The model was programmed using TensorFlow 2.5.0. PAR images were processed using OpenCV 4.5.4.60. The input images were grayscale images with a size of 256 × 256 pixels, and the value of each pixel was normalized from 0 to 1. The growth rate in the model was 12. Twelve feature maps were extracted in the bottleneck layers to decrease the model complexity and calculation computation.

The model was designed to classify whether the acetabular image included fractures. The training parameters were as follows: the batch size was set to 15 and the learning rate was initially set to 0.003 and was decreased every 10 epochs by 10%. An Adam optimizer was used, and a drop-box with a dropout rate of 0.2 was applied for training.

### Detecting tests for clinicians

We recruited five residents and five attending orthopedic surgeons with 1–4 and 5–8 years of acute trauma-related experience, respectively, for the diagnostic tests. The aim was to compare the diagnostic performance of clinicians with that of the DL model. Before testing, all 10 clinicians were instructed to focus on only one question, to indicate whether there was an AF. Then, the ROI images of the test set were automatically presented on a screen one by one at 5-s intervals to each participating clinician. The entire experiment was divided into two parts, and 280 images were captured simultaneously. This process was adopted to ensure fairness between DL and clinicians and between clinicians, even though the test process was different from that in real clinical settings. The ROI images from the validation set were also performed at one time as described above. Based on the test results, a potential misdiagnosed case (PMC) was defined as a fracture missed by five or more of the 10 clinicians. The sensitivity, specificity, positive predictive value, negative predictive value, and accuracy were calculated to evaluate the clinicians’ diagnostic performance.

### Statistical analysis

R 4.1.0 with “pROC” and “ggplot2” packages was used for statistical analyses and graphs. Continuous variables were compared using the Mann–Whitney *U* test, and categorical variables were compared using the chi-square and Fisher’s exact tests. The detection performance of the DL model was evaluated and compared using the receiver operating characteristic (ROC) curve and the area under the ROC curve (AUC) with 95% corresponding confidence intervals (CIs), which were estimated by bootstrapping (2,000 times). The optimal threshold value was determined using Youden’s J-statistic. McNemar’s test was used to compare the sensitivity and specificity between the DL model and clinicians. The statistical significance level was set at *p* < 0.05.

## Results

A total of 1,120 patients in a single trauma center were enrolled and randomly divided into development and test sets at a ratio of 3:1. Among the 1,120 selected patients, 8 had bilateral AFs. Therefore, 840 patients with 846 injured acetabula and 834 normal acetabula in the development set and 280 patients with 282 broken acetabula and 278 normal acetabula in the test set were obtained. Additionally, 420 type A, 412 type B, and 296 type C AFs were identified and classified into 1,128 ROI fracture images. A total of 86 patients with 86 injured acetabula containing 33 type A, 36 type B, and 17 type C AFs from two hospitals were enrolled. The patients’ demographic data are shown in Table 1. Regarding age, sex, injured side, mechanism of injury, and fracture classification, there were no statistical differences (all *p* > 0.05) between the test and validation sets.

**TABLE 1:** Patient demographics.

Variables	Internal single-center dataset			External validation dataset			*p*-value^a^
	Total	Development set	Test set	Total	Hospital A	Hospital B	
Patients	1,120	840	280	86	37	49	
Age, mean (range), years	46.41 (18–89)	46.53 (18–89)	46.06 (18–88)	49.05 (19–93)	49.54 (19–88)	48.67 (21–93)	0.202
Sex							0.192
Male	771	573	198	67	31	36	
Female	349	267	82	19	6	13	
Injured side							0.639
Left	489	378	111	39	20	19	
Right	623	456	167	47	17	30	
Both	8	6	2	0	0	0	
Mechanism of injury							0.598
Vehicle accident	587	458	129	38	15	23	
Fall	427	304	123	43	19	24	
Height < 2 m	160	116	44	16	8	8	
Height > 2 m	267	188	79	27	11	16	
Others	106	78	28	5	3	2	
Fracture type^b^							0.526
Type A	420	319	101	33	18	15	
Type B	412	304	108	36	14	22	
Type C	296	223	73	17	5	12	

^a^ *p* values are acquired by comparison between the test set and total of external validation set.

^b^ Fracture types are categorized according to the “Three-column classification of acetabular fractures.” Type A, B, and C represent fractures involving single one, two, and three columns of the acetabulum, respectively.

**FIGURE 2:**
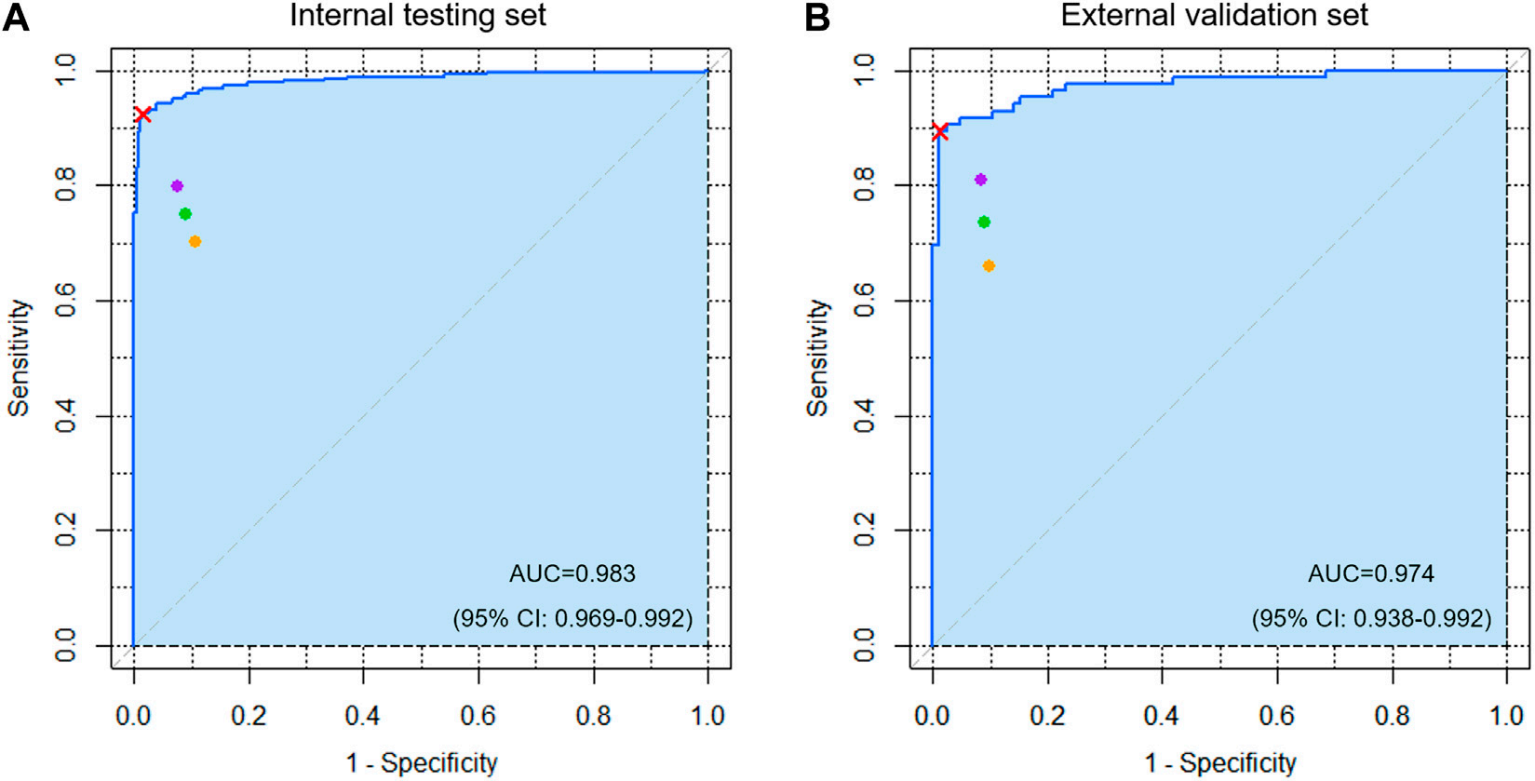
The receiver operating characteristic (ROC) curve of the detection algorithm for acetabular fractures in the **(A)** internal testing and **(B)** external validation sets. The cyan area indicates AUC. The red cross mark represents the performance on the probability cutoff value calculated by Youden’s J statistic. The purple, orange, and green spots represent the performance of five attending orthopedic surgeons, five residents, and ten clinicians, respectively.

For the test/validation set comprising 560/172 ROI images, the sensitivity, specificity, positive predictive value, negative predictive value, and accuracy of the DL detection model were 0.926/0.872, 0.978/0.988, 0.978/0.987, 0.928/0.885, and 0.952/0.930, respectively. The ROC curve with visual AUC is shown in Figure 2. The optimal threshold value was 0.637/0.256, and the Youden index was 0.911 (sensitivity, 0.926; specificity, 0.985)/0.883 (sensitivity, 0.895; specificity, 0.988) for the test/validation sets.

Table 2 shows the diagnostic performance of the DL model, clinicians, and their comparisons. The mean sensitivity, specificity, positive predictive value, negative predictive value, and accuracy of the 10 clinicians were 0.750/0.735, 0.909/0.909, 0.894/0.890, 0.786/0.774, and 0.829/0.822 for the test/validation sets, respectively. Moreover, the mean results of the five attending orthopedic surgeons are higher than that of the residents. However, the DL model performed better (*p* < 0.05) with respect to sensitivity and specificity, except for the sensitivity of one clinician and specificity of three clinicians in the test set and the sensitivity and specificity of four clinicians in the validation set.

**TABLE 2:** Performance comparison between the DL model and clinicians in detecting acetabular fractures.

	SEN		SPE		PPV		NPV		ACC	
	Internal	External	Internal	External	Internal	External	Internal	External	Internal	External
DL model	0.926	0.872	0.978	0.988	0.978	0.987	0.928	0.885	0.952	0.930
Attending orthopedics 1^c^	0.775	0.791	0.889	0.884	0.876	0.872	0.795	0.809	0.831	0.837
Attending orthopedics 2^b–d^	0.812	0.802	0.944	0.930	0.937	0.920	0.832	0.825	0.878	0.866
Attending orthopedics 3^b^	0.691	0.756	0.963	0.907	0.950	0.890	0.755	0.788	0.826	0.831
Attending orthopedics 4^c^	0.832	0.826	0.899	0.919	0.893	0.910	0.840	0.840	0.865	0.872
Attending orthopedics 5^a^ ^,^ ^c^ ^,^ ^d^	0.886	0.872	0.926	0.942	0.924	0.938	0.889	0.880	0.906	0.907
Mean	0.799	0.809	0.924	0.916	0.916	0.906	0.822	0.828	0.861	0.863
Resident 1^b^	0.731	0.686	0.944	0.919	0.930	0.894	0.776	0.745	0.837	0.802
Resident 2	0.795	0.756	0.889	0.919	0.879	0.903	0.811	0.790	0.842	0.837
Resident 3^d^	0.592	0.512	0.896	0.930	0.852	0.880	0.684	0.656	0.743	0.721
Resident 4^d^	0.667	0.640	0.926	0.953	0.901	0.932	0.733	0.726	0.795	0.797
Resident 5	0.721	0.709	0.815	0.791	0.798	0.772	0.742	0.731	0.767	0.750
Mean	0.701	0.660	0.894	0.902	0.872	0.871	0.749	0.727	0.797	0.781
Mean of above 10 surgeons	0.750	0.735	0.909	0.909	0.894	0.890	0.786	0.774	0.829	0.822

DL, deep learning; SEN, sensitivity; SPE, specificity; PPV, positive predictive value; NPV, negative predictive value; ACC, accuracy.

Results for the internal testing set.

^a^ Except for this clinician, all have significant difference (*p* < 0.05) between the DL model and a clinician in SEN.

^b^ Except for these clinicians, all have significant difference (*p* < 0.05) between the DL model and a clinician in SPE. Results for the external validation set.

^c^ Except for these clinicians, all have significant difference (*p* < 0.05) between the DL model and a clinician in SEN.

^d^ Except for these clinicians, all have significant difference (*p* < 0.05) between the DL model and a clinician in SPE.

**FIGURE 3:**
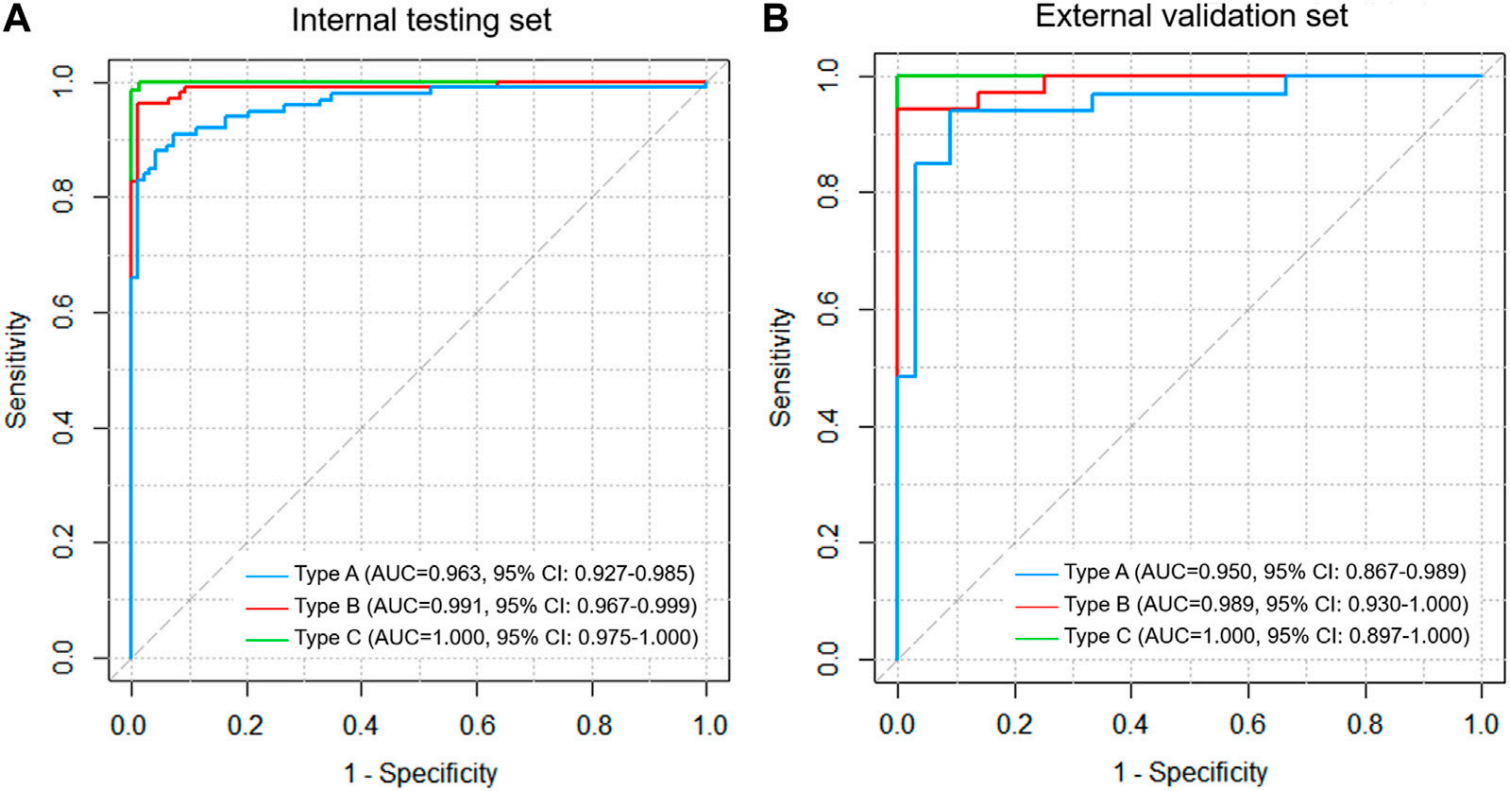
ROC curve analysis for types A, B, and C fractures based on the three-column classification of acetabular fractures in the **(A)** internal testing and **(B)** external validation set.

For types A, B, and C AFs, the DL model had the best diagnostic performance for type C fractures, with an AUC value of 1.000 (95% CI: 0.975–1.000)/1.000 (95% CI: 0.897–1.000); type A fracture was the worst, with an AUC value of 0.963 (95% CI: 0.927–0.985)/0.950 (95% CI: 0.867–0.989); and type B had an AUC value of 0.991 (95% CI: 0.967–0.999)/0.989 (95% CI: 0.930–1.000) in the test/validation sets (Figure 3). The gradient-weighted class activation mapping (Grad-CAM) method was applied to visualize the probable AF regions of different fracture types determined by the DL model (Supplementary Figure S1) (Selvaraju et al., 2017).

A total of 46 PMCs were misdiagnosed by at least five clinicians in the internal test and external validation sets, including five PMCs by five clinicians, eight PMCs by six clinicians, eight PMCs by seven clinicians, six PMCs by eight clinicians, ten PMCs by nine clinicians, and nine PMCs by ten clinicians. Of the 46 PMCs, 40 were type A fractures, 6 were type B, and none were type C. The DL model correctly recognized 26 of the 46 PMCs (Supplementary Figure S2).

## Discussion

In this study, we first developed a specific DL model to detect AFs on PARs with high sensitivity, specificity, and accuracy. According to our results, the diagnostic performance of the DL model was equal to or even better than that of the clinicians. Our model showed the highest performance in detecting the most complex type C fractures among all AF types. Furthermore, the model successfully identified 26 of the 46 cases in which the clinicians had a high probability of misjudgment. This shows that the model has a promising ability to detect PMCs.

The overall miss rate of fractures identified by clinicians on PARs was estimated to be 10% (Hakkarinen et al., 2012). However, unlike fractures in other areas, clinicians cannot easily identify Afs on PARs. In a recent study, only 42.4% (61/144) of AFs were detected by an attending trauma radiologist on PARs (Benjamin et al., 2022). Another study involving 129 patients showed that AFs were the most commonly missed trauma on PARs (Kessel et al., 2007). Similarly, our study found that clinicians missed up to 20% of the AFs cases. As mentioned above, diagnosis by humans is not very accurate. Nevertheless, we did not find previous specific studies on DL for detecting AFs on PARs, and insufficient image data on AFs could be one of the barriers to the development of an appropriate DL model. A 2019 study reported that AFs could be detected using traditional machine learning methods, achieving an accuracy of 80% in a test set containing as few as five cases (Castro-Gutierrez et al., 2019). Therefore, we conducted this study to prove that our DL model has great potential for diagnosing AFs.

Kuo et al. (2022) analyzed 37 studies of DL on X-rays for fracture detection from January 2018 to July 2020 and demonstrated that the median number of participants, the median size of development sets, the pooled sensitivity of test sets, and the pooled specificity of test sets were 1,169 (interquartile range, 425–2,417), 1,898 (interquartile range, 784–7,646), 92% (95% CI: 88–94), and 91% (95% CI: 88–93), respectively. Although both the number of participants and development set in this study were comparatively low, similar sensitivities and specificities were obtained. This indicates that the DL model is efficient.

AFs are classified based on radiographic imaging features. The Letournel-Judet criterion (Letournel, 1980) is the most widely applied classification of AFs, but it only covers 80% of them (Herman et al., 2018). The choice of an appropriate classification theory capable of covering and classifying all AFs is essential for presenting and discussing the test results in this study. Therefore, a more comprehensive three-column classification theory was adopted. All AFs were classified into three types, with higher grades indicating more areas involved and more severe fractures. The results showed that the DL model had excellent recognition ability for type C fractures, where injuries were severe and likely to be combined with other injuries, and low recognition ability for type A fractures, where injuries were relatively mild. This helps to quickly diagnose and save critically injured patients with AFs.

Clinician experience is negatively associated with fracture misdiagnosis rates; however, timely specialist consultations are usually unavailable (Williams et al., 2000). In this study, we defined the concept of PMC. PMC was neither summarized by the results of X-ray reports nor conferred by experienced specialists, but was based on the test results of 10 clinicians, which is closer to the real situation. DL algorithms are liable to misdiagnosis when abnormal features are subtle, even for experienced radiologists (Li et al., 2021). Although our model identified only 56.5% (26/46) of PMCs, it could still be a valuable solution compared with clinicians’ performance. Furthermore, a previous study demonstrated the utilization of the Grad-CAM algorithm in medical education and practice (Cheng et al., 2020b). Grad-CAM was used in this study to provide a reference for clinicians to assist in fracture diagnosis. The heat maps generated by Grad-CAM enable improved diagnostic accuracy with limited learning cases (Cheng et al., 2020b).

DenseNet was selected in this study because it has the main advantage of balancing the performance on the development, test and validation sets compared to some other common models (Cheng et al., 2020a). In addition, it has the following advantages: 1) a smaller number of parameters, 2) encouraging feature reuse, 3) easier network training, and 4) alleviating the problems of gradient vanishing and model degradation (Huang et al., 2017). Many successful presentations of the DenseNet model have been reported in the field of orthopedics, such as vertebral compression fractures (Monchka et al., 2021), proximal femoral fractures (Oakden-Rayner et al., 2022), distal radio-ulnar fractures (Kim et al., 2021), hip fractures (Krogue et al., 2020; Cheng et al., 2021), and hip osteoarthritis (von Schacky et al., 2020).

This study has some limitations. First, ROI images were generated by manual cropping, which resulted in subtle variations. Second, the diagnostic performance of clinicians may be underestimated because the procedure is not a real clinical scenario; for example, no information was available regarding patients’ clinical backgrounds. Lastly, intra-observer variability could be affected by the factors such as the surroundings and the clinician’s self-efficacy, which may affect the study findings. The algorithm should be externally validated by large numbers of cases in a multicenter prospective clinical setting in the future.

In conclusion, we developed a DL algorithm for detecting AFs, which achieved a diagnostic performance comparable to or even superior to that of clinicians. The algorithm has an excellent detection rate for severely injured type C fractures and is useful for detecting inconspicuous AFs. Future studies should conduct prospective controlled trials in real-world clinical settings to further demonstrate that DL can be used as a tool to aid clinicians in AF diagnosis.

## Data Availability

The original contributions presented in the study are included in the article/Supplementary Material, further inquiries can be directed to the corresponding author.
