# Supplementary material for: Development and validation of a deep learning-based model to distinguish acetabular fractures on pelvic anteroposterior radiographs
Source: Front Physiol. 2023 Apr 28;14:1146910. doi: 10.3389/fphys.2023.1146910 (PMC10176114; doi:10.3389/fphys.2023.1146910)
Supplement: Supplementary file 1 [file Table1.DOCX]

**Supplementary Material**

**Details of data augmentation**

The development set had 1680 original ROI images. Firstly, the brightness of all the images was increased and reduced by 20% respectively. After tuning the brightness and darkness, additional new 3360 images were archived. Then, to take account of disturbance caused by orientation, all these 5040 images were rotated with 10° both clockwise and anticlockwise, which provides additional 10080 images. Subsequently, we enlarged our development set with artificial gaussian noise and blur effect. 5040 images were filtered by a Gaussian kernel with a size of 3 by 3 and kernel standard deviation of 2. Besides, the same 5040 original images were also added with salt and pepper noise additionally. Finally, the dataset after the data augmentation process was with 25200 images. All the image augmentation and processing were carried out using Python with the package OpenCV-python.

Table S1. The Architecture for DenseNet-169

| Layer | Output size | Structure | |
| --- | --- | --- | --- |
| Convolution | 128×128 | 7×7 conv, 32, stride 2 | |
| Pooling | 64×64 | 3×3 max pool, stride 2 | |
| Dense Block 1 | 64×64 | 1×1 conv, 12  3×3 conv, 12 | ×6 |
|  |  |  |  |
| Transition Layer 1 | 64×64 | 1×1 conv, 104 | |
|  | 32×32 | 2×2 average pool, stride 2 | |
| Dense Block 2 | 32×32 | 1×1 conv, 12  3×3 conv, 12 | ×12 |
|  |  |  |  |
| Transition Layer 2 | 32×32 | 1×1 conv, 248 | |
|  | 16×16 | 2×2 average pool, stride 2 | |
| Dense Block 3 | 16×16 | 1×1 conv, 12  3×3 conv, 12 | ×32 |
|  |  |  |  |
| Transition Layer 3 | 16×16 | 1×1 conv, 632 | |
|  | 8×8 | 2×2 average pool, stride 2 | |
| Dense Block 4 | 8×8 | 1×1 conv, 12  3×3 conv, 12 | ×32 |
|  |  |  |  |
| Classification Layer | 1×1 | 8×8 global average pool | |
|  |  | 2D fully connected layer Softmax | |


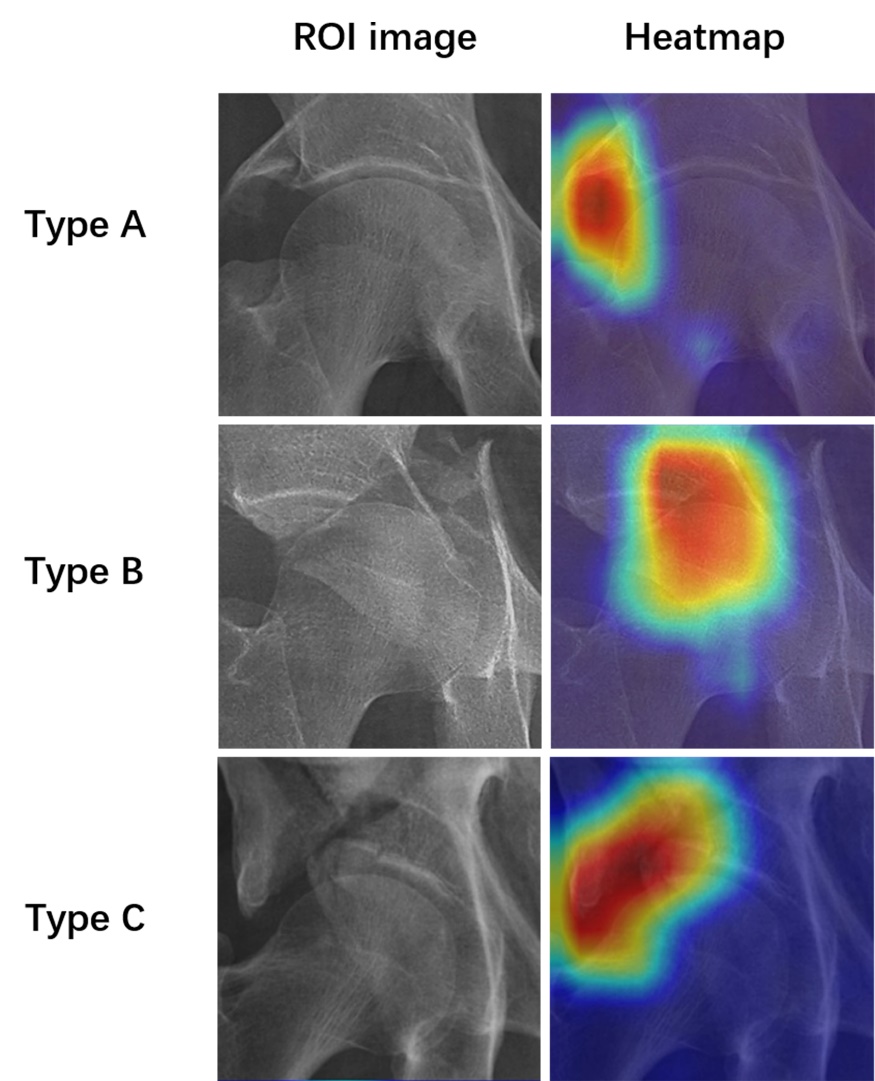


Fig. S1. Using gradient-weighted class activation mapping (Grad-CAM) technique to visualize probable fracture regions. The rows represent different types of acetabular fractures. The first column shows original ROI images. The second column shows prediction heat maps. The closer the color was to red, the more likely the DL model was to predict fractures.


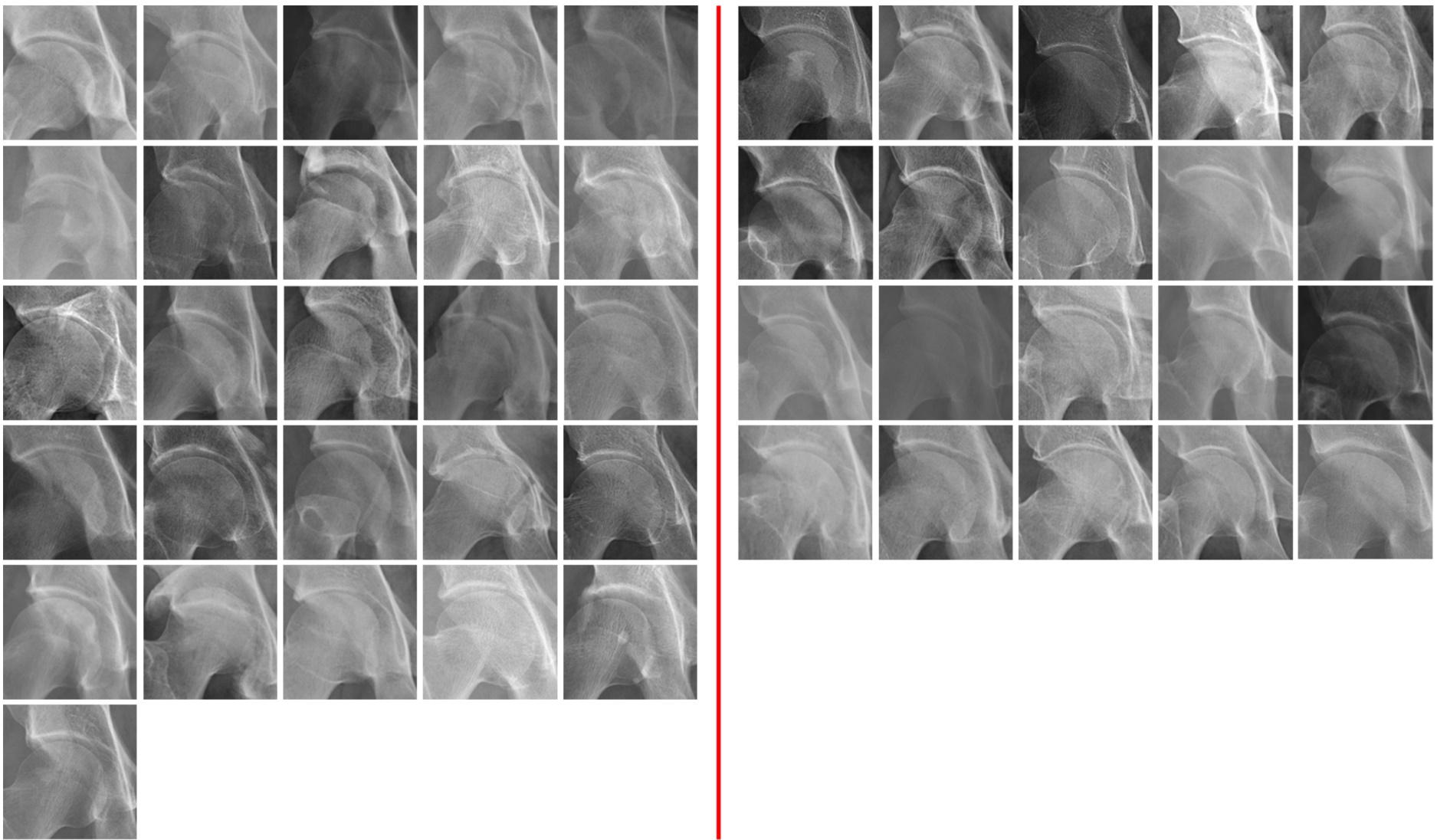


Fig. S2 Display of 46 PMCs in the internal testing and external validation sets (the left acetabulum has been flipped to right). Images on the left of the red line: 26 PMCs correctly diagnosed by the DL model. Images on the right of the red line: 20 PMCs misdiagnosed by the DL model. The last five images on the left and right belong to the external validation set. PMC: potential misdiagnosed case.
